# Changes in IL-16 Expression in the Ovary during Aging and Its Potential Consequences to Ovarian Pathology

**DOI:** 10.1155/2022/2870389

**Published:** 2022-04-26

**Authors:** Jessica Ramirez, Pincas Bitterman, Sanjib Basu, Animesh Barua

**Affiliations:** ^1^Department of Anatomy and Cell Biology, Rush University Medical Center, Chicago, IL 60612, USA; ^2^Departments of Pathology, and Obstetrics & Gynecology, Rush University Medical Center, Chicago, IL 60612, USA; ^3^Department of Internal Medicine, Rush University Medical Center, Chicago, IL 60612, USA; ^4^Departments of Anatomy and Cell Biology, Pathology and Obstetrics & Gynecology, Rush University Medical Center, Chicago, IL 60612, USA

## Abstract

Aging in females is not only associated with the changes in hormonal status but is also responsible for dysregulation of immune functions in various organs including ovaries. The goal of this study was to determine whether the expression of interleukin 16 (IL-16), a proinflammatory and chemoattractant cytokine, changes during ovarian aging, to determine factors involved in such changes in IL-16 expression, and to examine if changes in IL-16 expression during aging predisposes the ovary to pathologies. Ovarian tissues from premenopausal women (30-50 years old), women at early menopause (55-59 years old), and late menopause (60-85 years old) were used. In addition, tumor tissues from patients with ovarian high-grade serous carcinoma at early stage (*n* = 5) were also used as reference tissue for comparing the expression of several selected markers in aging ovaries. The expression of IL-16, frequency of macrophages (a source of IL-16) and expression of microRNA (miR) 125a-5p (a regulator of IL-16 gene) were performed by immunohistochemistry, immunoblotting, and gene expression assays. In addition, we examined changes in nuclear expression of IL-16 expression with regards to exposure to follicle-stimulating hormone (FSH) by in vitro cell culture assays with human ovarian cancer cells. The frequencies of IL-16 expressing cells were significantly higher in ovarian stroma in women at early and late menopause as compared with premenopausal women (*P* < 0.0001). Similar patterns were also observed for macrophages. Expression of miR-125a-5p decreased significantly (*P* < 0.001) with the increase in IL-16 expression during aging. Furthermore, expression of nuclear IL-16 increased remarkably upon exposure to FSH. Consequently, ovarian aging is associated with increased expression of IL-16 including its nuclear fraction. Therefore, persistent high levels of FSH in postmenopausal women may be a factor for enhanced expression of IL-16. Effects of increased nuclear fraction of IL-16 need to be examined.

## 1. Introduction

Aging in females is associated with a decrease in ovarian function including folliculogenesis and gonadal steroid production [1]. As the ovary ages, depletion in follicular recruitment and growth leads to the gradual withdrawal of ovarian steroid-induced negative feedback on the pituitary resulting in sustained high levels of circulatory gonadotrophin including follicle-stimulating hormone (FSH) [2, 3]. As estrogen is known to be involved in various physiological processes, decrease in its level during aging affects the growth and maintenance of its target organs [4–6]. On the other hand, sustained high levels of circulatory FSH during aging may perturb ovarian homeostatic balance facilitating the development of abnormal condition including chronic inflammation [7] and may be risk factor associated with ovarian cancer (OVCA). Moreover, estrogen is known to be associated with the enhancement and/or maintenance of immunity [8–11]. Thus, ovarian aging not only affects reproductive functions but it may also increase the susceptibility of ovarian tissues to chronic conditions. However, the effects of aging and its mechanism(s) in the ovary including chronic conditions are not well understood. This information is critically important for the prevention of various chronic abnormalities in the ovary specially in menopausal women.

Chronic inflammation and oxidative stress as it occurs in the ovary have been proposed as hallmarks of various pathological conditions including malignancy [12–14]. Ovarian tissues are exposed to various inflammatory factors as part of physiological processes like ovulation or infection by pathogens. Ovulation has been suggested as an inflammatory event [15]. With the release of egg during ovulation, ovarian surface epithelial cells at the site of ovulatory rupture and the fimbrial surface of the fallopian tube at the site of receiving the ovulated eggs are exposed to various toxic metabolites produced in the egg due to its metabolic processes [16]. Moreover, ovulatory injury leads to the influx of immune cells at the site of rupture in the ovarian surface and at the site of receiving of ovulated eggs in the fimbria. This results in localized inflammation and increased demand for oxygen by the accumulated immune Cell, facilitating the development of hypoxia and oxidative stress [17]. Thus, inflammation and oxidative stress are prevalent in the ovary and the fimbria of the fallopian tube. Furthermore, the oviduct is open to both external and internal environments through the vagina and fimbria, respectively, predisposing the reproductive tissues to external pathogens or internal toxic byproducts of various physiological processes. Close proximity of ovarian tissues with the fimbria increases the chance of gaining entrance of pathogens to the ovaries. In addition, food-borne pathogens from a perforated gastrointestinal tract may gain entrance to the ovary via the systemic circulation. Thus, the ovarian and fimbrial tissues are exposed to various inflammatory conditions due to frequent ovulation, external pathogens, and internal toxic byproducts as well as persistent high levels of circulatory FSH. Therefore, information on the effects of exposure to these agents will be helpful to prevent ovarian abnormalities during aging.

Ovarian tissues express many cytokines [18, 19]. Cytokines are proteins secreted by many cell types including immune cells, epithelial cells, fibroblasts, and stromal cells. Cytokines are involved in the regulation of cellular growth and differentiation, homeostasis, and immune functions in normal tissues as well as in pathological conditions including tumors [18]. In normal ovaries, interleukin- (IL-) 6 and TGF-*β* have been suggested to be involved in follicular development by preventing follicular atresia [20, 21]. IL-1 and TNF-*α* have been shown to be associated with inhibition of progesterone secretion and regression of the corpus luteum [22, 23]. Ovarian follicles have been reported to produce IL-8, while IL-11 was found in the follicular fluid [24, 25]. Unfortunately, most of the studies on cytokines on ovarian function are limited to premenopausal ovaries and studies on ovarian cytokines during aging including late menopausal stage ovaries are very scanty. Recent studies have shown increased expression of IL-16 in ovarian tumors [26, 27]. However, no information is available if persistent high levels of IL-16 expression are a risk factor to develop OVCA.

IL-16 is a proinflammatory cytokine and a chemotactic factor for other immune cells to the site of inflammation [28]. Frequent exposure of ovarian and fimbrial tissues to ovulatory insults, external and internal agents (pathogenic/metabolic), and increased levels of FSH may lead to chronic inflammation in these issues and may induce increased expression of IL-16. Chronic inflammation is a hallmark of cancer development. However, it is unknown if the expression of IL-16 increases during aging in ovaries and fimbria, and whether persistent high levels of IL-16 are associated with OVCA development. The goal of this study was to examine whether IL-16 expression increases in ovarian and fimbrial tissues during aging and whether such increase in IL-16 expression is associated with increased risk of OVCA in postmenopausal women.

## 2. Material and Methods

### 2.1. Clinical Specimens

Archived premenopausal and postmenopausal ovarian tissues from healthy/normal subjects and their blood samples were collected from the Department of Pathology Rush University Medical center, Chicago, IL. All specimens were collected under the Institutional Review Board (IRB) of the Rush University Medical Center approved protocol. These subjects underwent surgery for nonovarian cause. Ovarian tumor tissues (*n* = 3, ovarian high-grade serous carcinoma (HGSC), used as positive control) were collected from patients underwent surgery following the diagnosis of suspected ovarian mass.

Representative normal (healthy) specimens were divided into 3 groups, namely, premenopausal (30-50 years old, *n* = 8), early menopausal (55-59 years old, *n* = 7), and late menopausal (60-85 years old, *n* = 9).

### 2.2. Processing of Tissue Specimens

Ovarian tissues were processed for paraffin and/or frozen embedding, protein extraction, and gene expression studies. Briefly, upon receiving, tissues were washed with phosphate-buffered solution (PBS) and divided into four pieces including for paraffin and frozen sections, protein, and total RNA extraction. For paraffin embedding, tissue specimens were treated with neutral buffered formalin for 72 hours followed by washing with running water overnight, cut into blocks of desired sizes, dehydrated by treating with an ascending series of ethanol and xylene, and embedded in paraffin. The portion of fresh tissues for RNA extraction w**as** treated with RNA later (RNAlater™ Stabilization Solution, Thermofisher, Waltham, MA) and stored at -80°C for later use. For frozen sections, portions of fresh tissues were embedded in OCT compound (Miles Inc., Elkhart, IN) and snap-frozen in a mixture of methanol and solid carbon dioxide and stored at -80°C for later use. The portion of fresh tissues for protein extraction w**as** stored at -80°C for later use. Serum samples were separated from blood and stored at -80°C for further use.

### 2.3. Routine Stain

Sections (5 *μ*m) were made from paraffin embedded tissue blocks and stained with hematoxylin and eosin, examined under a light microscope by a board-certified pathologist for the presence any abnormality.

### 2.4. Immunohistochemical Studies

#### 2.4.1. Specimens

Paraffin-embedded 5 *μ*m sections were used to determine the expression of IL-16 (8 premenopausal ovaries, 7 early menopausal ovaries, and 9 late menopausal ovaries), macrophages (6 premenopausal ovaries, 6 early menopausal ovaries, and 5 late menopausal ovaries) and follicle-stimulating hormone receptor (FSHR) (*n* = 5 from each group) by immunohistochemistry.

#### 2.4.2. Antibodies and Reagents

Immunohistochemical examinations were performed using antihuman IL-16 (Abcam, Cambridge, MA, 1 : 100 dilution), antihuman FSHR (Abcam, Cambridge, MA, 1 : 100 dilution), or antihuman macrophages primary antibodies (Thermo Fisher Scientific, Waltham, MA, 1 : 100 dilution).

#### 2.4.3. Immunohistochemistry (IHC)

IHC was carried out as reported earlier [26] using primary antibodies and other reagents as per the instructions of the manufacturers. Briefly, sections were deparaffinized with xylene and a graded series of alcohol followed by washing briefly in deionized (DI) water. Antigens on the section were unmasked by heating the sections with sodium citrate-containing antigen retrieval solution. Sections were then cooled at room temperature in phosphate-buffered solution (PBS) followed by blocking of endogenous peroxidase using 0.03% H_2_O_2_-containing methanol at ice-cold condition. Sections were then rinsed in PBS and treated with normal horse serum for 15 min to block endogenous nonspecific bindings using VECTASTAIN ABC Kit (Vector laboratories, INC., Burlingame, CA). Sections were then incubated overnight at 4°C with primary antibodies mentioned above. Following washing 5 min X 3 with PBS, sections were incubated at room temperature with secondary antibodies and avidin/biotinylated enzyme complex (VECTASTAIN ABC Kit (Vector laboratories, INC., Burlingame, CA)) one hour each and 5 min X 3 washing with PBS in between. After washing with PBS, immunoreactions on sections were visualized by incubating them with a chromogen DAB solution (3.3′-diaminobenzidine, DAB-Peroxidase Substrate Kit—Vector laboratories, INC., Burlingame, CA) while examining under a light microscope. Sections were then washed in running water followed by counterstaining with hematoxylin. After rinsing in water, sections were then dehydrated with a graded series of alcohol and xylene, mounted with mounting media, covered with cover slip, and oven dried. Control (negative) staining was carried out by omitting primary antibodies, and no staining was observed in these sections (Supplementary figure 1).

#### 2.4.4. Counting of Immunopositive Cells or Intensity of Immunostaining

Frequency of immunopositive cells was counted, or intensities of immunostaining were determined using a light microscope attached to a computer-assisted digital imaging software (MicroSuite, version 5; Olympus Corporation, Tokyo, Japan). Counting was performed by two individuals blinded of the age or pathology of the subjects/patients. For IL-16 or macrophages, immunopositive cells in 3-5 areas with highest population in a section at 40X magnification were counted, and average of them was expressed as the frequency of IL-16-expressing cells or macrophages in 20,000 *μ*m^2^ area of the tissue as reported earlier [26]. Similarly, the intensities of FSHR expression were expressed in 20,000 *μ*m^2^ area as an arbitrary value as reported earlier with little modification [29].

#### 2.4.5. Western Blotting

Proteins were extracted from tissue samples as reported earlier [26]. Briefly, tissue specimens were homogenized using a Polytron homogenizer (Brinkman Instruments, Westbury, NY). Homogenized samples were then centrifuged, supernatants were collected, and protein concentrations from supernatants were measured using Bradford BioRad Protein Assay (Bio-Rad, Hercules, CA) method as reported earlier [26]. Protease inhibitor was added, and samples were stored at -80°C for later use. Three samples from each age group (including 30-50-, 55-59-, 60-69-, and 70-85-year old subjects) as well as OVCA patients were selected randomly for immunoblotting, and each sample was examined two times (2X) in immunoblotting. Briefly, a panel of 4 samples (tissue extracts), one from each age group, and a positive control (indicated below) was examined in each immunoblot. In gel electrophoresis, the same amount of protein (50 g) for each sample was loaded. Proteins were separated by one-dimensional electrophoresis as reported earlier [26], and separated proteins were transferred to the membrane. Immunoblotting of membranes was performed using anti-IL-16 mentioned above as primary antibodies (at 1 : 1000 dilution) and anti-rabbit HRP as secondary antibody. Immunoreactions on the membrane were visualized as chemiluminescence products (Super Dura West substrate; Pierce/Thermo Fisher, Rockford, IL). Images were captured by Quantity One software using Chemidoc XRS (Bio-Rad, Hercules, CA) system according to the manufacturer's recommendation as reported previously [26]. Images of 3 immunoblots were selected randomly for analysis. Intensity of signals of IL-16 protein expression in immunoblotting was determined from the images using the analysis® getIT! Software (Olympus Soft Imaging Solutions Corporation, Lakewood, CO). Signal intensities were quantified as arbitrary values and reported as mean + SEM in 20,000 *μ*m^2^ area. An ovarian HGSC specimen was used as positive control for IL-16 protein expression while *β*-actin protein was used as housekeeping protein.

#### 2.4.6. Gene Expression Studies (Quantitative Real-Time Polymerase Chain Reaction)

Expression of IL-16 gene and its regulator microRNA miR-125a-5p was examined in representative specimens (8 premenopausal ovaries, 6 early menopausal ovaries, and 4 late menopausal ovaries) by quantitative real-time polymerase chain reaction (qRT-PCR). Total RNA was extracted from all specimens using TRIzol reagent (Invitrogen, Carlsbad, CA) according to the manufacturer's recommendation. RNA was then measured at an optical density (OD) of 260 nm and an OD of 260/280 nm absorbance ratio ≥1.7 was used to evaluate the purity, as previously reported [30].

The expression of IL-16 messenger RNA (mRNA) and miR-125a-5p in normal ovaries and fimbriae was measured by quantitative real-time polymerase chain reaction (qRT-PCR). The human specific IL-16 primer (QT00075138) designed by QuantiTech and miR-125a-5p designed by Applied Biosystems (Foster City, CA) were used for qRT-PCR analyses. *β*-Actin was used as housekeeping gene in qRT-PCR experiments. Gene expression amplification was determined using the method of the differences (*δ*) in cycle threshold (*Δ*Ct) for IL-16 mRNA and miR-125a-5p according to the manufacturer's recommendation. Subtracting *Δ*Ct from each group from the average *Δ*Ct determined the *ΔΔ*Ct. 2^–*ΔΔ*Ct^ was used to calculate the fold change in the differences in IL-16 mRNA and miR-125a-5p expression levels.

#### 2.4.7. Treatment of Cells with FSH and Cell Fractionation

Human ovarian surface epithelium (OSE) cells were a kind gift from Dr. Hazel Lum, PhD, Department of Pharmacology, Rush University Medical Center. The OSE cells were grown in ovarian epithelium cell medium (OEpiCM) (ScienCell Research Laboratories, Carlsbad, CA) supplemented with 1% ovarian epithelial cell growth supplement (OEpiCGS, ScienCell Research Laboratories, Carlsbad, CA), 5% fetal bovine serum (FBS) (SAFC Biosciences Inc., Lenexa, KS), and 1% of antibiotic solution made of penicillin and streptomycin (P/S) (ScienCell Research Laboratories, Carlsbad, CA). OSE cells were then plated in 100 mm dishes with approximately 2.5 × 10^6^ cells and were treated in triplicate with 4 uL of human recombinant FSH (Sigma-Aldrich, St. Louis, MO) mixed with 16 uL of media per well for 24 hours. After incubation, media was collected and saved. Cell fractionation was performed using the Cell Fractionation Kit (Abcam, Cambridge, UK) according to the manufacturer's recommendation. Cells were rinsed with PBS, trypsinized, and harvested (pellet). Pellet containing FSH-treated or untreated (control) were then resuspended in 1X Buffer A to 6.6 × 10^6^ cells/mL and diluted by 1,000-fold using Buffer B. Cells were incubated at room temperature for 7 minutes with constant mixing, followed by centrifugation at 5,000 × g for 1 minute at 4°C. Pellet was removed and saved, while supernatant was then removed and centrifuged at 10,000 × g for 1 minutes at 4°C. The final supernatant contains fraction C (Cytosol). The saved pellet was then resuspended in Buffer A, diluted in Buffer C, and incubated at room temperature for 10 minutes with constant mixing. The suspension was centrifuged at 5,000 × g for 1 minute at 4°C. Pellet was removed and saved, while supernatant was then removed and centrifuged at 10,000 × g for 1 minutes at 4°C. The final supernatant contains fraction M (mitochondrial). The saved pellet was resuspended in Buffer A, containing fraction N (nuclear).

### 2.5. Statistical Analysis

Differences in the frequency of IL-16-expressing cells or macrophages or in the intensities of FSHR expression during aging were assessed by ANOVA and unpaired *t*-tests. Differences in the signal intensity of IL-16 protein expression in immunoblotting among different age groups were also determined by ANOVA and unpaired *t*-tests. All reported *P* values were a two-sided where *P* < 0.05 was considered significant. Statistical analysis was performed using the Prism GraphPad software.

## 3. Results

**Figure 1:**
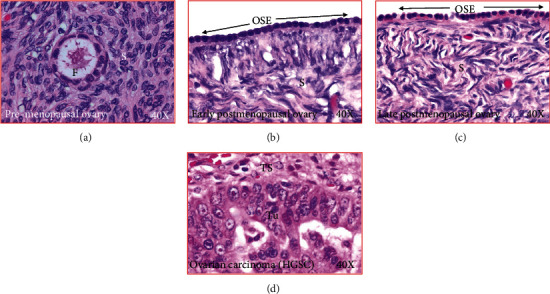
Microscopic presentations of healthy ovaries and ovary with cancer. (a) Section of an ovary from a premenopausal subject. An embedded follicle is seen in the ovarian stroma. (b) Section of an ovary from a healthy early postmenopausal woman showing no embedded follicle in the stroma. The ovarian surface layer is seen to be composed of rounded or flat-type of epithelial cells. (c) Section of an ovary from a healthy late postmenopausal woman showing no embedded follicle in the stroma. The ovarian surface layer is seen to be composed of rounded or flat-type of epithelial cells. (d) Section of an ovarian high-grade serous carcinoma (HGSC) at late stage. OSE: ovarian surface epithelial layer; S: stroma; TS: tumor stroma; Tu: tumor; 40×: magnification.

**Figure 2:**
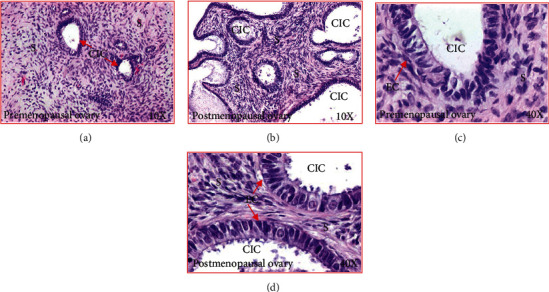
Cortical inclusion cysts (CIC) in healthy ovaries in pre- and postmenopausal women. (a) Section of an ovary from a premenopausal woman at low magnification (10×). Few CICs are seen in the ovarian stroma. (b) Section of an ovary from a healthy woman at late postmenopausal stage (low magnification, 10×). Compared with premenopausal woman, more CICs are seen in the ovarian stroma. (c) Section of an ovary from a healthy premenopausal woman (presented in (a)) showing a CIC in the stroma at high magnification (40×). The CICs consist of a single layer of tube-like or columnar-like epithelial cells. (d) Section of an ovary from a healthy late postmenopausal woman (presented in (b)) showing CICs in the stroma (at high magnification, 40×). As seen in the premenopausal ovary, CICs are consisted with a single layer of tube-like or columnar-like epithelial cells. EC: epithelial cells; S: stroma.

**Figure 3:**
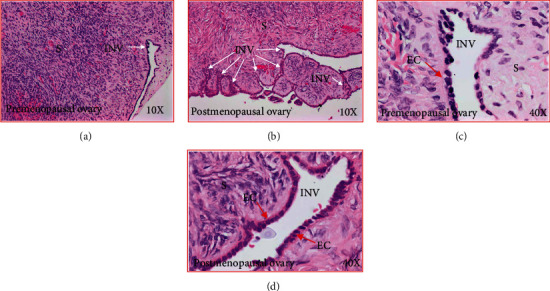
Stromal invaginations (INV) in healthy ovaries in pre- and postmenopausal women. (a) Section of an ovary from a premenopausal woman showing an INV in the stroma at low magnification (10×). (b) Section of an ovary from a healthy woman at late postmenopausal stage (low magnification, 10×). Compared with premenopausal woman, many INVs of different sizes and shapes are seen in the ovarian stroma. (c) Section of an ovary from a healthy premenopausal woman (presented in (a)) showing an INV in the stroma at high magnification (40×). The INV consists of a single layer of rounded or occasionally tube-like epithelial cells. (d) Section of an ovary from a healthy late postmenopausal woman (presented in (b)) showing an INV in the stroma (at high magnification, 40×). As seen in the premenopausal ovary, the INV is consisted with a single layer of rounded or occasionally tube-like epithelial cells. EC: epithelial cells; S: stroma.

Ovarian H&E sections from premenopausal subjects showed preantral follicles were embedded in the stroma, while no follicle was observed in sections from early and late menopausal women (Figure 1). Cortical inclusion cysts (CIC) (Figure 2) and ovarian surface invaginations (INV) (Figure 3) were observed in ovarian sections from pre- and postmenopausal women. Differences in the histomorphology of these features among pre- and postmenopausal women at early and late stages were not observed (Figures 2 and 3). However, compared with premenopausal, CICs and INVs were more frequent in late menopausal ovaries (Figures 2 and 3).

### 3.1. Localization of IL-16 in Ovaries

**Figure 4:**
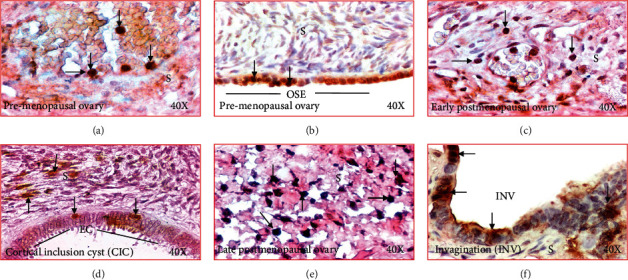
Changes in immunolocalization of IL-16 in human ovaries during aging. (a) Section of a premenopausal ovary showing few immunopositive IL-16-expressing cells in ovarian stroma. (b) Section of an ovary from a subject at early stage of menopause. Compared with premenopausal, more immunopositive IL-16-expressing cells are seen in the stroma. (c) Section of an ovary from a subject at late stage of postmenopause. Many immunopositive IL-16-expressing cells are localized in the stroma. (d) Section of a premenopausal ovary showing a few IL-16-expressing cells in ovarian surface epithelial (OSE) layer. (e) Section of an ovary from a subject at late menopausal stage showing IL-16 expression by the epithelial cells (EC) in a cortical inclusion cyst (CIC) in ovarian stroma. (f) Section of an ovary from a subject at late menopausal stage showing IL-16 expression by cells of stromal invagination (INV). Compared with OSE and CIC, more IL-expressing cells are seen in the epithelial cells (EC) in INV. S: stroma. Magnification = 40×.

Immunopositive IL-16 expressing cells were localized in the ovarian surface layer as well as in the stroma in all specimens examined including ovarian tissues from premenopausal, early, and late menopausal women (Figure 4). IL-16-expressing cells showed multiple morphologic sizes and shapes including small, medium, and large as well as rounded, elongated, and irregular (Figures 4(a)–4(c)). Epithelial cells in OSE, CIC, or INVs also showed positivity for IL-16 expression (Figures 4(d)–4(f)).

**Figure 5:**
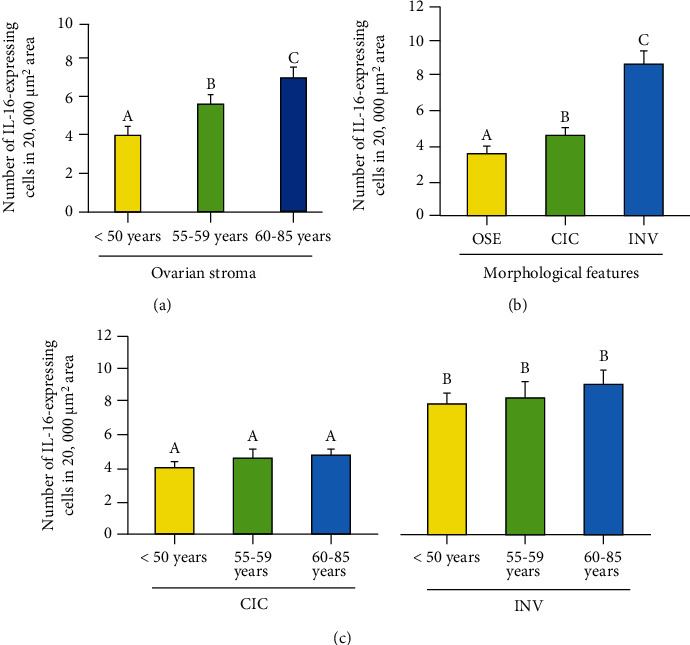
Changes in the frequency of IL-16-expressing cells in the ovary during aging. (a) Frequency of IL-16-expressing cells in the ovarian stroma in premenopausal and postmenopausal ovaries. Compared with premenopausal, the frequency of IL-16-expressing cells was significantly higher (*P* < 0.001) in subjects at early stage of menopause (55-59 years old) and increased further in subjects at late stage of menopause (60-85 years old). (b) Population of IL16-expressing cells in the ovarian surface epithelial (OSE) layer, cortical inclusion cysts (CICs), and stromal invaginations (INVs) in postmenopausal ovaries. Compared with OSE, the frequency of IL-16-expressing cells was significantly higher in CICs (*P* < 0.0001) and increased further in INVs (*P* < 0.0001). (c) Frequency of IL-16-expressing cells in CICs and INVs in different age groups. Significant differences were not observed in the frequency of IL-16 expressing cells in CICs among different age groups (five ovaries were randomly selected from each age group including premenopausal, early menopausal, and late menopausal stages and examined) (left panel, *P* < 0.72). Similarly, differences were not observed in the population of IL-16-expressing cells in INVs among different age groups (five ovaries were randomly selected from each age group including premenopausal, early menopausal, and late menopausal stages and examined) (right panel, *P* < 0.36). *y*-axis shows mean ± SEM in 20,000 mm^2^ area of the tissue and bars with different letters are significantly different. Details of statistical analysis are mentioned in materials and method section of the main text.

The frequency of IL-16-expressing cells in the ovarian stroma of premenopausal subjects was 4.0 ± 0.2 cells in 20,000 *μ*m^2^ area of the tissue. However, it increased significantly (*P* < 0.0001) to 5.9 ± 0.2 cells in 20,000 *μ*m^2^ area of the tissue in women at an early menopausal stage (Figure 5(a)) and increased even further (7.0 ± 0.3 in 20,000 *μ*m^2^ area of the tissue) with aging in subjects at a late menopausal stage (*P* < 0.0001) (Figure 5(a)).

INVs and CICs are formed by OSE cells as a result of ovulatory ruptures. The frequency of IL-16-expressing cells in OSE was 3.6 ± 0.2 in 20,000 *μ*m^2^ of the tissue (Figure 5(b)). Compared with OSE, the frequency of IL-16-expressing cells was significantly higher (*P* < 0.0001) in the epithelial layer of CICs (4.85 ± 0.1 in 20,000 *μ*m^2^ of the tissue) and increased further (12.66 ± 0.2 in 20,000 *μ*m^2^ of the tissue) in the epithelial cells in INVs (*P* < 0.0001). Furthermore, significant differences in the frequency of IL-16-expressing cells in CICs among different age groups (4.7 ± 0.3, 5.0 ± 0.4, and 4.8 ± 0.1mean ± SEM in 20,000 *μ*m^2^ area of the tissue in premenopausal, early postmenopausal, and late postmenopausal groups, respectively) were not observed (*P* < 0.72) (Figure 5(c), left panel). Similarly, differences were not observed in the frequency of IL-16-expressing cells in INVs in ovaries in different age groups (12.6 ± 0.2, 12.3 + 0.5, and 13.1 ± 0.4mean ± SEM in 20,000 *μ*m^2^ area of the tissue in premenopausal, early postmenopausal, and late postmenopausal groups, respectively) (*P* < 0.36) (Figure 5(c), right panel).

### 3.2. IL-16 Expression Observed by Immunoblotting

**Figure 6:**
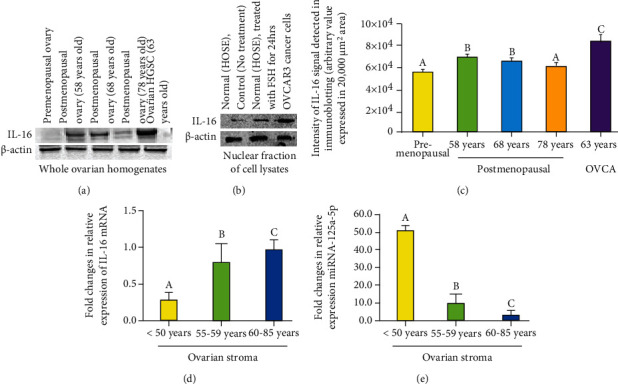
Changes in IL-16 protein and gene expression in the ovary during aging. (a) Western blot showing IL-16 expression during ovarian aging. (a) Changes in IL-16 protein expression in the ovary during aging. A very weak or faint immunoreactive band for IL-16 protein is seen in the ovary of a 38-year-old woman. Compared with premenopausal women, expression of IL-16 was stronger in 58- and 68-year-old postmenopausal women. However, expression of IL-16 protein was lower in 78-year-old postmenopausal ovary. As expected, expression of IL-16 protein was strongest in patient with ovarian high-grade serous carcinoma (HGSC). *β*-Actin protein was used as housekeeping protein. (b) Enhancement in IL-16 expression in response to exposure to follicle-stimulating hormone (FSH). Nuclear fraction in untreated (control) normal human ovarian surface epithelial (OSE) cells showed relatively weaker expression for IL-16. Compared with untreated, OSE cells treated with FSH for 24 hours showed stronger expression of IL-16. Similar patterns of expression were also observed in ovarian HGSC cells (OVCAR3). *β*-Actin protein was used as housekeeping protein. (c) Changes in the intensity of IL-16 expression in ovaries during aging and in ovarian tumor were detected by Western blotting. Each bar represents the mean intensity of signal for IL-16 expression (arbitrary values, reported as mean ± SEM in 20,000 *μ*m^2^ area) in three immunoblot assays. Bars with different letters are significantly different (compared to “a,” “b” is significant with *P* < 0.005, compared to “b,” “c” is significant with *P* < 0.03). (d, e) Changes in the relative expression of IL-16 gene and its regulator miR-125a-5p in ovaries during aging. (d) Fold changes in expression of IL-16 gene in the ovaries during aging including premenopausal and postmenopausal ovaries. Compared with premenopausal, the expression of IL-16 gene was significantly higher (*P* < 0.001) in subjects at early stage of menopause and increased further in subjects at late stage of menopause. (e) Fold changes in the expression of miR-125a-5p in the ovaries in premenopausal and postmenopausal women. Compared with premenopausal, the expression of miR-125a-5p decreased significantly (*P* < 0.001) in subjects at early stage of menopause and reduced further in subjects at late stage of menopause. Bars with different letters are significantly different. *y*-axis shows mean ± SEM of fold changes in IL-16 and miR-125a-5p gene expression, and bars with different letters are significantly different. Details of statistical analysis are mentioned in materials and method section of the main text.

Immunoblotting studies showed a band of approximately ~60 kDa for IL-16 in all specimens examined with different intensities (Figure 6(a)). In immunoblotting, a weak or faint band for IL-16 protein was detected in specimens from premenopausal subjects (Figure 6(a)). In contrast, subjects in the early menopausal group showed strong expression for IL-16 in immunoblotting which was stronger in subjects at the late menopausal stage (Figure 6(a)). However, the intensity of IL-16 protein expression was weaker in subjects older than 70 years. Nuclear fraction in untreated (control) normal OSE cells showed relatively weaker expression for IL-16 (Figure 6(b)). Compared to untreated counterparts, OSE cells treated with FSH for 24 hours showed stronger expression of IL-16 (Figure 6(b)). Similar patterns of expression were also observed in ovarian HGSC cells (OVCAR3).

Compared to premenopausal (5.9 × 10^4^ ± 0.8 × 10^3^ in 20,000 *μ*m^2^ area), the intensity of signal of IL-16 expression in immunoblotting was significantly higher in 58-year-old postmenopausal woman (1.13 times, *P* < 0.0003) (Figure 6(c)). Significant differences were not observed in the intensity of signals of IL-16 expression between 58-year and 68-year-old postmenopausal women (*P* < 0.46). However, compared to 68-year-old (6.6 × 10^4^ ± 0.9 × 10^3^ in 20,000 *μ*m^2^ area), the intensity of signals of IL-16 expression decreased significantly in 78-year-old postmenopausal woman (6.1 × 10^4^ ± 0.79 × 10^3^ in 20,000 *μ*m^2^ area) (*P* < 0.01). Nevertheless, as expected, the intensity of signals of IL-16 expression was highest in patient with ovarian HGSC (approximately 1.21 times more than 58 years old postmenopausal woman, *P* < 0.03) (Figure 6(c)). However, significant differences were not observed in the expression of *β*-actin among different groups (*P* < 0.97) (Supplementary figure 2).

Compared with untreated normal OSE cells (1.14 × 10^6^ ± 2.5 × 10^4^ in 20,000 *μ*m^2^ area of the blot), the nuclear fraction of the OSE cells treated with FSH for 24 hours showed a significant increase in IL-16 expression (1.28 × 10^6^ ± 1.1 × 10^4^ in 20,000 *μ*m^2^ area of the blot) (*P* < 0.03). Similarly, significantly higher expression of IL-16 (1.66 × 10^6^ ± 4.8 × 10^3^ in 20,000 *μ*m^2^ area of the blot) (*P* < 0.001) was detected in the nuclear fraction of OVCAR3 ovarian cancer cells. However, significant differences were not observed in *β*-actin expression among different groups, including normal OSE cells untreated or treated with FSH for 24 hours and OVCAR3 cells (1.67 × 10^6^ ± 0.059 × 10^2^ in 20,000 *μ*m^2^ area of the blot, 1.65 × 10^6^ ± 7.78 × 10^3^ in 20,000 *μ*m^2^ area of the blot and 1.64 × 10^6^ ± 6.76 × 10^3^ in 20,000 *μ*m^2^ area of the blot, respectively) (Supplementary figure 3).

### 3.3. Expression of IL-16 Gene and Its Regulatory MicroRNA

Expression of IL-16 gene was detected by qRT-PCR in all specimens examined (Figure 6(d)). Compared to subjects in premenopausal stage, expression of IL-16 gene increased significantly in subjects at early menopausal stage (*P* < 0.001) and even further in subjects at the late menopausal stage (Figure 6(d)).

Gene expression studies showed that increase in IL-16 gene expression during aging was inversely associated with expression of its regulatory microRNA, miR-125a-5p (Figure 6(e)). Compared with premenopausal subjects, expression of miR-125a-5p was significantly lower in women at an early menopausal stage and decreased further in subjects at the late menopausal stage (Figure 6(e)). Overall, gene expression studies supported an inverse relation between the expression of IL-16 gene and its regulator miR-125a-5p during ovarian aging.

### 3.4. Localization of Macrophages

**Figure 7:**
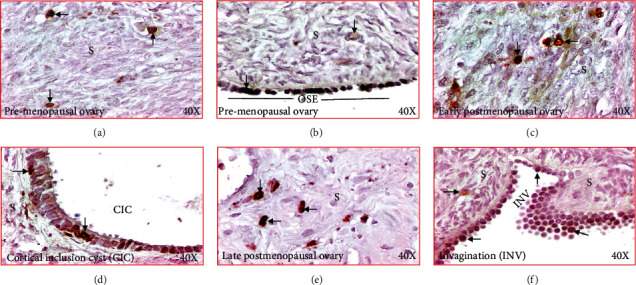
Changes in immunolocalization of macrophages in human ovaries during aging. (a) Section of a premenopausal ovary showing few immunopositive macrophages in ovarian stroma. (b) Section of an ovary from a subject at early stage of menopause. Compared with premenopausal, more macrophages are seen in the stroma. (c) Section of an ovary from a subject at late stage of menopause. Many macrophages are seen in the stroma. (d) Section of a premenopausal ovary showing a few macrophages in ovarian surface epithelial (OSE) layer. (e) Section of an ovary from a subject at late postmenopausal stage showing IL-16 expression by the epithelial cells (EC) in a cortical inclusion cyst (CIC) in ovarian stroma. (f) Section of an ovary from a subject at late menopausal stage showing macrophages localized in the stromal invagination (INV). S: stroma. 40×: magnifications. Arrows indicate examples of immunopositive macrophages.

**Figure 8:**
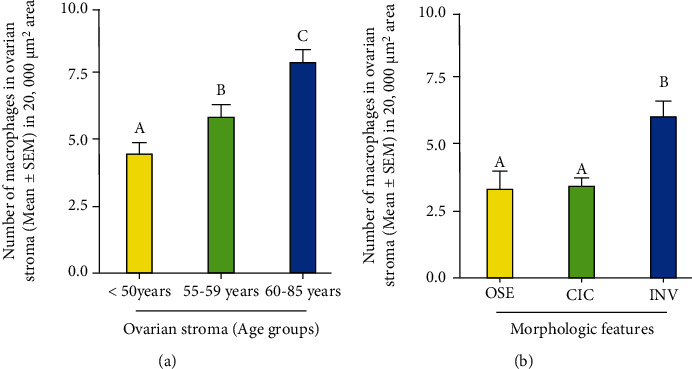
Changes in the frequency of macrophages in human ovaries during aging. (a) Frequency of macrophages in the ovarian stroma in premenopausal and postmenopausal ovaries. Compared with premenopausal, the frequency of macrophages was significantly higher (*P* < 0.001) in subjects at early stage of menopause (55-59 years old) and increased further in subjects at late stage of menopause (60-85 years old). (b) Population of macrophages in cortical inclusion cysts (CICs) and stromal invaginations (INVs) in menopausal ovaries. Compared with CIC, the frequency of macrophages was significantly higher in INVs (*P* < 0.0001). *y*-axis shows mean ± SEM of macrophages in 20,000 *μ*m^2^ area of the tissue, and bars with different letters are significantly different. Details of statistical analysis are mentioned in materials and method section of the text.

Macrophages have been suggested to be a source of IL-16 production. Immunohistochemical studies detected macrophages in the stroma as well as in the vicinity of CICs and INVs, two structures formed after ovulatory rupture (Figures 7(a)–7(f)). The frequency of macrophages in the ovarian stroma in women at premenopausal stage was 4.8 ± 0.1 in 20,000 *μ*m^2^ area of the tissue (Figure 8(a)). In contrast, the frequency of macrophages was significantly higher in the ovarian stroma in women at early (5.4 ± 0.2 in 20,000 *μ*m^2^ area of the tissue) (*P* < 0.05) and late menopausal stages (6.9 ± 0.3 in 20,000 *μ*m^2^ area of the tissue) (*P* < 0.0001) (Figure 8(a)). Furthermore, compared with CICs (3.4 ± 0.2 in 20,000 *μ*m^2^ area of the tissue), the frequency of macrophages (5.8 ± 0.4 in 20,000 *μ*m^2^ area of the tissue) was significantly higher in ovarian INVs (*P* < 0.0001) (Figure 8(b)).

### 3.5. Expression of Follicle-Stimulating Hormone Receptors (FSHR)

**Figure 9:**
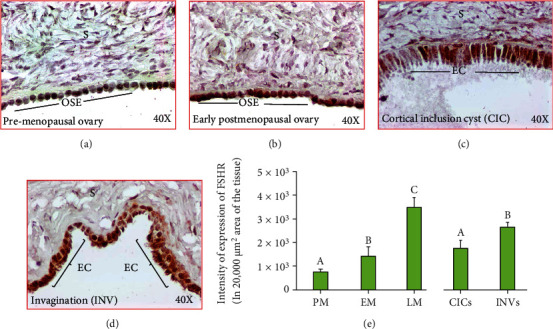
Expression of follicle-stimulating hormone receptor (FSHR) in ovaries during aging. (a)–(d): (a) Section of an ovary from a premenopausal subject showing a weak or moderate expression for FSHR by the ovarian surface epithelial (OSE) cells. (b) Section of an ovary from an early-stage postmenopausal subject showing strong expression for FSHR by the ovarian surface epithelial (OSE) cells. (c, d) Ovarian sections from healthy late postmenopausal women showing expression of FSHR by the epithelial cells (EC) in cortical inclusion cyst (CIC) and stromal invagination (INV), respectively. Compared with CIC, stromal INV showed stronger staining for FSHR expression. S: stroma; magnification: 40×. (e) Compared with premenopausal subjects, the intensities of FSHR expression were significantly (*P* < 0.04) higher in early menopausal women and increased further in late-stage menopausal women (*P* < 0.0001) (e). Furthermore, compared with CICs, the intensity of FSHR expression was significantly higher in INVs (*P* < 0.004) (e). *y*-axis shows mean ± SEM (*n* = 5 for each group) in 20,000 *μ*m^2^ area of the tissue, and bars with different letters are significantly different. Details of statistical analysis are mentioned in materials and method section of the main text.

Menopause is one of the most remarkable physiological changes in human females and is associated with the increase in circulatory levels of FSHR. In this study, immunohistochemical studies detected FSHR expression in all ovarian specimens with different degrees of signal intensities in respective of their menopausal stage (Figure 9). In premenopausal ovaries, OSE cells showed weak to moderate intensities for FSHR expression (Figure 9(a)). In contrast, the expression of FSHR was stronger in the OSE cells in subjects at early postmenopausal stage (Figure 9(b)). Similar patterns were also seen in subjects at late menopausal stages. Furthermore, compared with CIC (Figure 9(c)), the intensity of FSHR expression was stronger in INV (Figure 9(d)).

Compared to premenopausal (7.89 × 10^3^ ± 1.2 × 10^3^ in 20,000 *μ*m^2^ area of the tissue) subjects, the intensities of FSHR expression were significantly (*P* < 0.04) higher in early menopausal women (14.25 × 10^3^ ± 3.73 × 10^3^ in 20,000 *μ*m^2^ area of the tissue) and increased even further in late stage (35.07 × 10^3^ ± 3.81 × 10^3^ in 20,000 *μ*m^2^ area of the tissue, *P* < 0.0001) (Figure 9(e)). Furthermore, compared to CICs (17.79 × 10^3^ ± 3.12 × 10^3^ in 20,000 *μ*m^2^ area of the tissue), the intensity of FSHR expression was significantly higher in INVs (26.4 × 10^3^ ± 1.89 × 10^3^ in 20,000 *μ*m^2^ area of the tissue, *P* < 0.0047) (Figure 9(e)).

## 4. Discussion

This is the first study reporting an increase in the frequency of IL-16-expressing cells in ovaries during aging in women at postmenopausal stage. This study also showed significant increase in the frequency of IL-16-expressing cells in ovarian stromal invaginations (INVs) but not in cortical inclusion cysts (CICs), a structure formed following ovulation in the ovaries. Furthermore, this study also showed that increase in IL-16 gene expression was associated with the decrease in its regulatory microRNA miR-125a-5p during aging. The increase in the frequency of IL-16-expressing cells during aging was associated with the increase in the frequency of macrophages and persistent high levels of FSH in postmenopausal women. In addition, FSH treatment of normal ovarian cells showed increased expression of nuclear IL-16. Overall, the results of this study suggest that ovarian aging is associated with prevalence of chronic stress and inflammation, and two risk factors reported to be associated with ovarian pathologies including malignancy.

This study showed that expression of IL-16 including the frequency of IL-16 expressing cells in the ovary increased significantly during aging suggesting the prevalence of chronic inflammation in ovaries in late menopausal stage subjects. Classical inflammation requires coordination among different cell types and their secretions that mediate responses against deleterious stimuli [31]. However, inflammation in ovarian tissues during aging does not present the features of classical inflammation as it is not associated with infection, widespread tissue injury, or autoimmune conditions. In contrast, age-associated inflammation in ovaries is local and may be due to metabolic imbalances [13, 32] caused by agents including hormones during aging. Aging in females is associated with the decrease in ovarian functions and cessation of synthesis of ovarian steroids including estrogen [3]. It is possible that decrease in estrogen may be involved in the development of chronic inflammation in menopausal ovaries during aging.

Estrogen has been implicated as an anti-inflammatory agent as it has been shown to suppress the secretion of inflammatory cytokines including IL-6 and TNF-*α* by macrophages and dendritic cells [33]. Furthermore, circulatory levels of TNF-*α*, IL-1, and IL-6 have been reported to be increased in women at late menopausal stage, and their levels decreased significantly in response to hormone replacement therapy (HRT) [34]. Thus, it is possible that lack of estrogen in the ovaries in women at late menopausal stage may be a reason for the increased levels of IL-16 expression in aging ovaries. Alternatively, it is possible that persistent high levels of FSH in late menopausal stage women may be a factor for high levels of IL-16 expression.

Absence of negative feedback (due to the lack of estrogen) leads to the persistent high levels of FSH in women at late menopausal stage. Macrophages have been suggested to be a source of IL-16 synthesis [35]. Macrophage stimulating factor (MCSF) is an important cytokine which is involved in the regulation of proliferation [36], differentiation, and migration of tissue macrophages as well as is important for the maintenance of ovarian function [37]. Increasing concentrations of FSH have been shown to stimulate the expression of MCSF receptor mRNA suggesting the enhancement in MCSFR expression by FSH [38]. This action of FSH has been reported to be inhibited by estrogen treatment. Thus, it is possible that persistent high levels of FSH in postmenopausal women might be a reason of increased IL-16 production by macrophages in aging ovaries. This assumption is further supported by the results of this study that the frequencies of macrophages were higher in women at late menopausal stage than premenopausal women. However, specific targets of FSH in aging ovaries in the context of IL-16 secretion are not fully understood.

This study showed, in addition to OSE cells, epithelial cells in CICs and INVs were positive for FSHR expression suggesting that INVs and CICs are also targets for FSH. This study further showed that compared with OSE and CICs, INVs showed stronger expression of FSHR. Thus, INVs might be a predilection site for a chronic inflammation due to persistent exposure to high FSH. INVs and CICs are features formed by the ovarian surface epithelial layer in the ovary following ovulations. This study also showed that compared to OSE cells, the frequency of IL-16-expressing cells was higher in CICs and highest in INVs. Thus, it is possible that CICs and INVs might be invaded by immune cells including macrophages (in response to ovulatory insults) which may be a source of increased expression of IL-16 in these tissues. Alternatively, chronic inflammation may be prevalent in CICs and INVs due to their persistent exposure to FSH as observed by the increased expression of FSHR in these tissues. This assumption is supported by one of the observations of this study that treatment of normal OSE cells with human recombinant FSH for 24 hours resulted in remarkable increase in nuclear expression of IL-16 with similarities in patterns of expression by OVCAR3 cancer cell lines. However, how the expression of IL-16 increases at molecular levels is not known.

MicroRNAs are endogenously synthesized short noncoding RNA molecules [39] which bind to the 3′untranslated region (UTR) of target genes and play important roles in gene regulation at the posttranscriptional level, thereby, inhibit or reduce the translation of respective target genes [40]. In this study, the levels of IL-16 expression during aging increased while the expression of its regulatory miR-125a-5p decreased significantly in women at late menopausal stage. Although specific reason(s) involved in the decrease in miR-125a-5p leading to the increase in IL-16 gene expression is not known, it is possible that changes in hormonal milieu during aging in postmenopausal women may be a factor. Because menopause is associated with the cessation of estrogen production by the ovary [41] and high persistent levels of circulating FSH, it is possible that either the lack of estrogen or high levels of FSH might have a role in the suppression of IL-16-gene regulating microRNA miR-125a-5p. Estradiol treatment has been reported to enhance expression of miR-125b [42]. Thus, it is possible that increased expression of IL-16 gene in aging ovaries might be due to the decrease in its regulatory microRNA miR-125a-5p expression because of the cessation of estrogen synthesis in postmenopausal women.

Overall, IL-16, a pro-inflammatory and chemotactic cytokine, is produced by a variety of cells including immune cells and epithelial cells of different organs [43–45]. IL-16 has been implicated in several cancers including OVCA [26, 46]. OVCA in most cases is a malignancy of postmenopausal women and the median age of OVCA incidence is 63 years. Longstanding unresolved oxidative stress and chronic inflammation have been suggested as predisposing factors for malignancy including OVCA [13, 14]. Stromal INVs and CICs are structures formed by the ovarian surface layer following ovulation and have been shown to be a predilection site for malignant transformation [47]. Increased expression of IL-16 in these structures as observed in this study suggests the prevalence of chronic inflammation in these structures. Moreover, deletions in the chromosome 19 with approximately 60% loss of heterozygosity have been reported to be associated with OVCA [48, 49], and interestingly, miR-125a-5p is localized in the 19q13.41 locus of this chromosome. The consequence of increase in nuclear expression of IL-16 due to FSH exposure is not known. It is possible that, following enhancement in expression as a result of chronic oxidative stress due to the persistent exposure to FSH, IL-16 may translocate to the nucleus and lead to the formation of mutagenic DNA adducts. Previous reports suggest the formation of mutagenic DNA-adducts due to oxidation of DNA bases which may lead to malignancy [50]. Thus, dysregulation in miR-125a-5p during aging may be a reason for increased expression of IL-16 in postmenopausal women and may also increase the risk of developing OVCA as it is a disease of postmenopausal women.

In conclusion, results of this study suggest that expression of IL-16, a proinflammatory and chemotactic cytokine, increases during aging in the ovaries in postmenopausal women. This increase in IL-16 expression was localized in CICs and INVs, the two structures in the ovary formed following ovulation and are sites with risk of malignant transformation. Moreover, increase in IL-16 expression was associated with its regulator microRNA miR-125a-5p, also a tumor suppressor microRNA. Thus, chronic inflammation in the ovaries in postmenopausal women may predispose them to ovarian pathology including malignant transformation in ovaries.

## Data Availability

All data supporting the conclusions of this article are included in the article.

## Supplementary Materials

Supplementary Materials Figure S-1: Control (negative) staining for antibodies used in the present study. Figure S-2: Intensity of *β*-actin protein expression in healthy ovarian tissues and ovarian high grade serous carcinoma. Figure S-3: (a) Intensity signal of IL-16 expression in the nuclear fraction of untreated normal OSE cells, the nuclear fraction of OSE cells treated with FSH for 24 hours, and OVCAR3 cells. (b) Intensity of *β*-actin protein expression in the nuclear fraction of normal OSE cells untreated or treated with FSH for 24 hours, and OVCAR3 cells.
