# Supplementary material for: Changes in IL-16 Expression in the Ovary during Aging and Its Potential Consequences to Ovarian Pathology
Source: J Immunol Res. 2022 Apr 26;2022:2870389. doi: 10.1155/2022/2870389 (PMC9053759; doi:10.1155/2022/2870389)
Supplement: Supplementary Materials — Figure S-1: Control (negative) staining for antibodies used in the present study. Figure S-2: Intensity of β-actin protein expression in healthy ovarian tissues and ovarian high grade serous carcinoma. Figure S-3: (a) Intensity signal of IL-16 expression in the nuclear fraction of untreated normal OSE cells, the nuclear fraction of OSE cells treated with FSH for 24 hours, and OVCAR3 cells. (b) Intensity of β-actin protein expression in the nuclear fraction of normal OSE cells untreated or treated with FSH for 24 hours, and OVCAR3 cells. [file 2870389.f1.zip › Supplemental figures _2.pptx]

## Slide 1
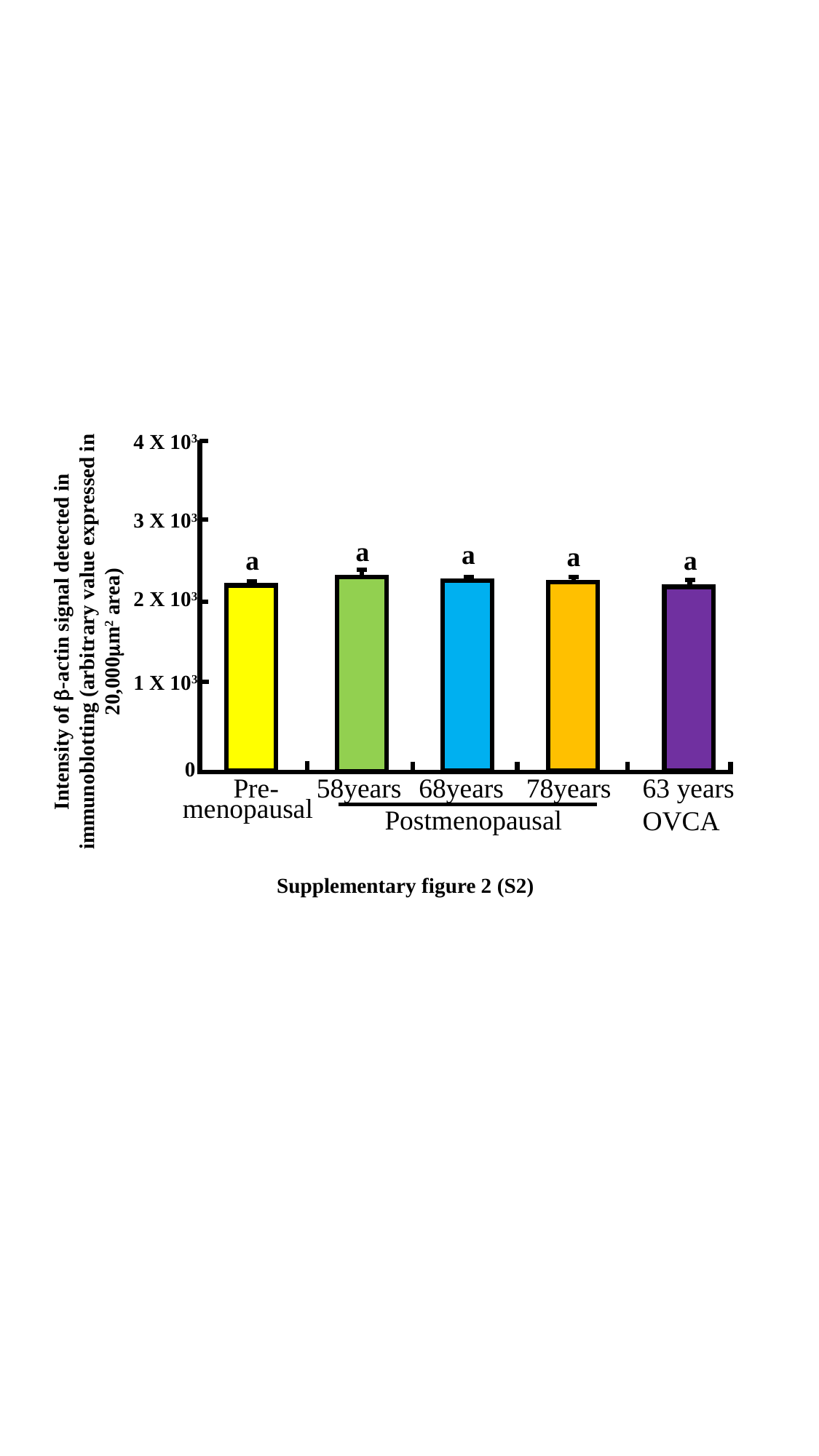

4 X 103
3 X 103
a
a
a
a
a
2 X 103
Intensity of b-actin signal detected in immunoblotting (arbitrary value expressed in 20,000mm2 area)
1 X 103
0
Pre-
58years
68years
78years
63 years OVCA
menopausal
Postmenopausal
Supplementary figure 2 (S2)
