# Supplementary material for: Changes in IL-16 Expression in the Ovary during Aging and Its Potential Consequences to Ovarian Pathology
Source: J Immunol Res. 2022 Apr 26;2022:2870389. doi: 10.1155/2022/2870389 (PMC9053759; doi:10.1155/2022/2870389)
Supplement: Supplementary Materials — Figure S-1: Control (negative) staining for antibodies used in the present study. Figure S-2: Intensity of β-actin protein expression in healthy ovarian tissues and ovarian high grade serous carcinoma. Figure S-3: (a) Intensity signal of IL-16 expression in the nuclear fraction of untreated normal OSE cells, the nuclear fraction of OSE cells treated with FSH for 24 hours, and OVCAR3 cells. (b) Intensity of β-actin protein expression in the nuclear fraction of normal OSE cells untreated or treated with FSH for 24 hours, and OVCAR3 cells. [file 2870389.f1.zip › Supplemental figures _3.pptx]

## Slide 1
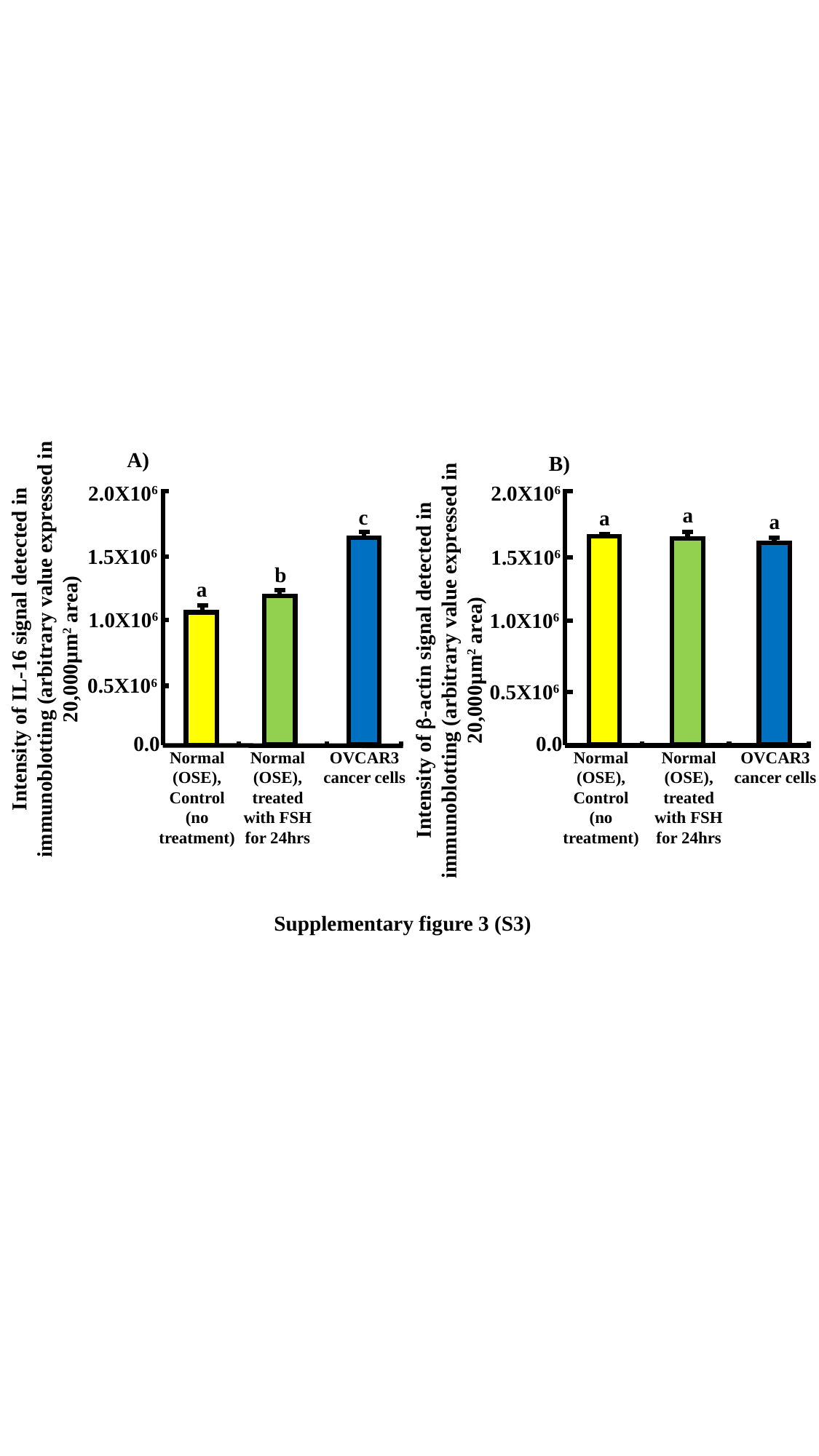

A)
2.0X106
c
1.5X106
b
a
1.0X106
0.5X106
0.0
Normal (OSE), Control (no treatment)
Normal (OSE), treated with FSH for 24hrs
OVCAR3 cancer cells
B)
2.0X106
a
a
a
1.5X106
1.0X106
0.5X106
0.0
Normal (OSE), Control (no treatment)
Normal (OSE), treated with FSH for 24hrs
OVCAR3 cancer cells
Intensity of IL-16 signal detected in immunoblotting (arbitrary value expressed in 20,000μm2 area)
Intensity of b-actin signal detected in immunoblotting (arbitrary value expressed in 20,000μm2 area)
Supplementary figure 3 (S3)
